# (1*RS*,2*RS*,3*SR*,5*RS*,7*RS*)-2,5-Dichloro-8-oxabicyclo­[5.1.0]octan-3-ol

**DOI:** 10.1107/S1600536811013304

**Published:** 2011-04-16

**Authors:** Yasin Çetinkaya, Abdullah Menzek, Tuncer Hökelek

**Affiliations:** a Department of Chemistry, Atatürk University, 25240 Erzurum, Turkey; b Department of Physics, Hacettepe University, 06800 Beytepe, Ankara, Turkey

## Abstract

In the title compound, C_7_H_10_Cl_2_O_2_, the seven-membered ring displays a chair conformation. In the crystal, the hy­droxy H atom is equally disordered over two orientations, and links with an adjacent mol­ecule *via* an O—H⋯O hydrogen bond in both cases. Weak inter­molecular C—H⋯O hydrogen bonding is also a feature of the crystal structure.

## Related literature

For background to *syn*-bis-epoxides, see: Balcı (1981 ▶); Akbulut *et al.* (1987 ▶); Menzek & Balcı (1993 ▶); Saraçoğlu *et al.* (1999 ▶). For background to unsaturated bicyclic endopexide, see: Menzek *et al.* (2005 ▶). For background to epoxide and bis-epoxide, see: Şengül *et al.* (2008 ▶).
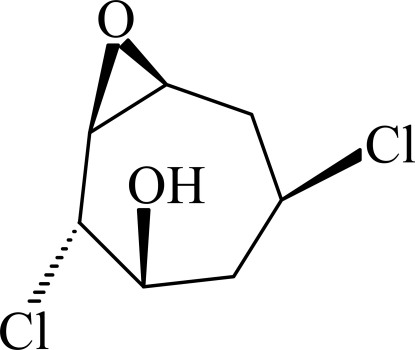


## Experimental

### 

#### Crystal data

C_7_H_10_Cl_2_O_2_
*M* *_r_* = 197.05
Orthorhombic, 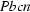

*a* = 21.9202 (5) Å
*b* = 9.9343 (3) Å
*c* = 8.1005 (2) Å
*V* = 1763.98 (8) Å^3^
*Z* = 8
Mo *K*α radiation
μ = 0.68 mm^−1^
*T* = 294 K
0.32 × 0.20 × 0.15 mm

#### Data collection

Rigaku R-AXIS RAPID-S diffractometer
Absorption correction: multi-scan (Blessing, 1995 ▶) *T*_min_ = 0.845, *T*_max_ = 0.900
32813 measured reflections
1801 independent reflections
1267 reflections with *I* > 2σ(*I*)
*R*_int_ = 0.110

#### Refinement

*R*[*F* ^2^ > 2σ(*F* ^2^)] = 0.088
*wR*(*F* ^2^) = 0.190
*S* = 1.18
1801 reflections
115 parameters
2 restraints
H atoms treated by a mixture of independent and constrained refinement
Δρ_max_ = 0.28 e Å^−3^
Δρ_min_ = −0.34 e Å^−3^

### 

Data collection: *CrystalClear* (Rigaku/MSC, 2005 ▶); cell refinement: *CrystalClear*; data reduction: *CrystalClear*; program(s) used to solve structure: *SHELXS97* (Sheldrick, 2008 ▶); program(s) used to refine structure: *SHELXL97* (Sheldrick, 2008 ▶); molecular graphics: *ORTEP-3 for Windows* (Farrugia, 1997 ▶) and *Mercury* (Macrae *et al.*, 2006 ▶); software used to prepare material for publication: *WinGX* (Farrugia, 1999 ▶) and *PLATON* (Spek, 2009 ▶).

## Supplementary Material

Crystal structure: contains datablocks I, global. DOI: 10.1107/S1600536811013304/xu5182sup1.cif

Structure factors: contains datablocks I. DOI: 10.1107/S1600536811013304/xu5182Isup2.hkl

Additional supplementary materials:  crystallographic information; 3D view; checkCIF report

## Figures and Tables

**Table 1:** Hydrogen-bond geometry (Å, °)

*D*—H⋯*A*	*D*—H	H⋯*A*	*D*⋯*A*	*D*—H⋯*A*
O2—H2*A*⋯O2^i^	0.83 (11)	1.96 (11)	2.746 (7)	159 (12)
O2—H2*B*⋯O2^ii^	0.85	1.84	2.692 (8)	174
C2—H21⋯O1^iii^	0.97	2.43	3.398 (7)	172

Symmetry codes: (i) 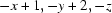; (ii) 
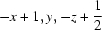
; (iii) 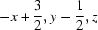.

**Fig. 1.**
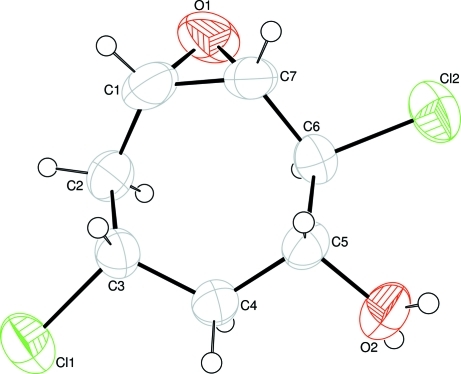
The molecular structure of the title molecule with the atom-numbering scheme. Displacement ellipsoids are drawn at the 40% probability level.

**Fig. 2.**
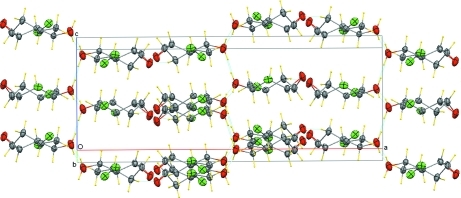
A view of the crystal packing of the title compound. The O-H···O and C-H···O hydrogen bonds are shown as dashed lines.

## supplementary crystallographic information

### Comment

Unsaturated bicyclic endopexides are important compounds for versatile chemical transformations in organic chemistry. In these endoperoxides, diradicals formed by thermal cleavage of the weak O-O bonds react to give the syn-bis-epoxides (Balcı, 1981; Akbulut *et al.*, 1987; Menzek & Balcı, 1993; Saraçoğlu *et al.*, 1999).

Unsaturated bicyclic endopexide, (1), (Scheme 1) was synthesized by the literature method (Menzek *et al.*, 2005). Reaction of endoperoxide, (1), by heating at 453 (5) K gave a mixture of products (Scheme 1). The title compound, (2), was isolated from these mixtures. The other products were not identified. According to the NMR data of dichloride, (2), it was not easy to establish the exact configuration of the molecule. Therefore, the exact structure of dichloride, (2), was determined by X-ray single crystal analysis.

To rationalize the formation of dichloride, (2), we propose the following reaction mechanism as favourable mechanism (Scheme 1). Bis-epoxide, (3), is produced via diradicals formed by thermal cleavage of the weak O-O bonds. HCl formed by elimination from reaction products such as (3) can attack bis-epoxide, (3), to give intermediate (4). Cl^-^ can attack intermediate (4) to give dichloride, (2), as a nucleophile. An epoxide or bis-epoxide ring (Şengül *et al.*, 2008) may be opened by as a nucleophile.

In the title compound, the seven-membered ring A (C1-C7) is, of course, not planar. The planar moieties B (O1/C1/C7), C (C3-C5), D (C2/C3/C5/C6) and E (C1/C2/C6/C7) are oriented at dihedral angles of B/C = 68.75 (46)°, B/D = 13.84 (28)°, B/E = 74.48 (32)°, C/D = 54.91 (42)°, C/E = 6.00 (42)° and D/E = 60.65 (30)°.

In the crystal, intermolecular O—H···O and C—H···O hydrogen bonds link the molecules into a three-dimensional network (Table 1 and Fig. 2).

### Experimental

For the preparation of the title compound, a mixture of endoproxide (0.5 g, 2.6 mmol) and benzene (5 ml) was placed into a test tube, sealed under vacuum and heated at 453 (5) K for 3 d. After cooling to room temperature, the solvent was evaporated. The residue was submitted to column chromatography (silica gel, 90 g) with AcOEt/hexane (1:6) as eluant. Dichloride (yield: 0.056 g, 9%, m. p. 366-368 K) and a mixture of unidentified products were obtained. Dichloride was crystallized from ethyl acetate/hexane (1:1) as colorless block crystals.

### Refinement

H1, H7, H21, H22 and H41, H42 atoms were positioned geometrically with C—H = 0.98 and 0.97 Å, respectively, and constrained to ride on their parent atoms, with *U*_iso_(H) = 1.2*U*_eq_(C). The remaining H-atoms were located in a difference Fourier map and refined isotropically. The H atom of the OH group was disordered over two orientations. During the refinement process, the disordered H2A and H2B atoms were refined with equal occupancies.

### Crystal data

**Table:**

C_7_H_10_Cl_2_O_2_	*F*(000) = 816
*M**_r_* = 197.05	*D*_x_ = 1.484 Mg m^−^^3^
Orthorhombic, *P**b**c**n*	Mo *K*α radiation, λ = 0.71073 Å
Hall symbol: -P 2n 2ab	Cell parameters from 4978 reflections
*a* = 21.9202 (5) Å	θ = 2.3–26.4°
*b* = 9.9343 (3) Å	µ = 0.68 mm^−^^1^
*c* = 8.1005 (2) Å	*T* = 294 K
*V* = 1763.98 (8) Å^3^	Block, colorless
*Z* = 8	0.32 × 0.20 × 0.15 mm

### Data collection

**Table:**

Rigaku R-AXIS RAPID-S diffractometer	1801 independent reflections
Radiation source: fine-focus sealed tube	1267 reflections with *I* > 2σ(*I*)
graphite	*R*_int_ = 0.110
ω scans	θ_max_ = 26.4°, θ_min_ = 2.3°
Absorption correction: multi-scan (Blessing, 1995)	*h* = −27→27
*T*_min_ = 0.845, *T*_max_ = 0.900	*k* = −12→12
32813 measured reflections	*l* = −9→10

### Refinement

**Table:**

Refinement on *F*^2^	Primary atom site location: structure-invariant direct methods
Least-squares matrix: full	Secondary atom site location: difference Fourier map
*R*[*F*^2^ > 2σ(*F*^2^)] = 0.088	Hydrogen site location: inferred from neighbouring sites
*wR*(*F*^2^) = 0.190	H atoms treated by a mixture of independent and constrained refinement
*S* = 1.18	*w* = 1/[σ^2^(*F*_o_^2^) + (0.020*P*)^2^ + 5.1831*P*] where *P* = (*F*_o_^2^ + 2*F*_c_^2^)/3
1801 reflections	(Δ/σ)_max_ < 0.001
115 parameters	Δρ_max_ = 0.28 e Å^−^^3^
2 restraints	Δρ_min_ = −0.34 e Å^−^^3^

### Special details

**Table:**

Geometry. All esds (except the esd in the dihedral angle between two l.s. planes) are estimated using the full covariance matrix. The cell esds are taken into account individually in the estimation of esds in distances, angles and torsion angles; correlations between esds in cell parameters are only used when they are defined by crystal symmetry. An approximate (isotropic) treatment of cell esds is used for estimating esds involving l.s. planes.
Refinement. Refinement of F^2^ against ALL reflections. The weighted R-factor wR and goodness of fit S are based on F^2^, conventional R-factors R are based on F, with F set to zero for negative F^2^. The threshold expression of F^2^ > 2sigma(F^2^) is used only for calculating R-factors(gt) etc. and is not relevant to the choice of reflections for refinement. R-factors based on F^2^ are statistically about twice as large as those based on F, and R- factors based on ALL data will be even larger.

### Fractional atomic coordinates and isotropic or equivalent isotropic displacement parameters (Å^2^)

**Table:**

	*x*	*y*	*z*	*U*_iso_*/*U*_eq_	Occ. (<1)
Cl1	0.59049 (9)	0.41547 (16)	0.1157 (2)	0.0883 (6)	
Cl2	0.62620 (9)	1.07361 (16)	0.1076 (2)	0.0841 (6)	
O1	0.7389 (2)	0.8227 (5)	0.1601 (7)	0.1017 (17)	
O2	0.5199 (2)	0.8930 (5)	0.0928 (7)	0.0941 (16)	
H2A	0.511 (7)	0.945 (11)	0.017 (12)	0.090*	0.50
H2B	0.5050	0.8950	0.1900	0.090*	0.50
C1	0.7119 (3)	0.7145 (7)	0.0682 (10)	0.084 (2)	
H1	0.7361	0.6806	−0.0247	0.101*	
C2	0.6766 (2)	0.6091 (6)	0.1650 (9)	0.0732 (18)	
H21	0.7003	0.5271	0.1751	0.088*	
H22	0.6672	0.6421	0.2748	0.088*	
C3	0.6182 (3)	0.5825 (6)	0.0692 (8)	0.0604 (15)	
H3	0.628 (3)	0.578 (7)	−0.042 (9)	0.10 (2)*	
C4	0.5669 (2)	0.6810 (5)	0.1042 (8)	0.0610 (14)	
H41	0.5303	0.6467	0.0516	0.073*	
H42	0.5595	0.6806	0.2223	0.073*	
C5	0.5748 (2)	0.8269 (6)	0.0507 (8)	0.0582 (14)	
H5	0.580 (2)	0.832 (5)	−0.066 (7)	0.057 (16)*	
C6	0.6294 (3)	0.8926 (5)	0.1265 (7)	0.0558 (13)	
H6	0.636 (2)	0.873 (5)	0.231 (7)	0.066 (18)*	
C7	0.6885 (2)	0.8499 (6)	0.0502 (8)	0.0703 (17)	
H7	0.6993	0.8954	−0.0531	0.084*	

### Atomic displacement parameters (Å^2^)

**Table:**

	*U*^11^	*U*^22^	*U*^33^	*U*^12^	*U*^13^	*U*^23^
Cl1	0.1032 (13)	0.0592 (9)	0.1024 (14)	−0.0091 (8)	−0.0189 (11)	0.0044 (9)
Cl2	0.1150 (14)	0.0573 (9)	0.0799 (11)	−0.0025 (9)	0.0052 (10)	−0.0046 (8)
O1	0.067 (3)	0.095 (4)	0.143 (5)	−0.021 (3)	−0.030 (3)	0.028 (3)
O2	0.067 (3)	0.084 (3)	0.132 (5)	0.024 (2)	0.023 (3)	0.012 (3)
C1	0.049 (3)	0.097 (5)	0.107 (6)	0.011 (3)	0.009 (4)	0.011 (5)
C2	0.055 (3)	0.069 (4)	0.096 (5)	0.014 (3)	−0.016 (3)	0.010 (3)
C3	0.066 (4)	0.052 (3)	0.063 (4)	0.001 (3)	0.001 (3)	−0.005 (3)
C4	0.049 (3)	0.058 (3)	0.075 (4)	0.001 (2)	−0.009 (3)	−0.004 (3)
C5	0.050 (3)	0.064 (3)	0.061 (4)	0.008 (3)	−0.001 (3)	0.004 (3)
C6	0.066 (3)	0.049 (3)	0.052 (3)	0.001 (2)	−0.009 (3)	0.000 (3)
C7	0.052 (3)	0.075 (4)	0.084 (4)	−0.006 (3)	0.009 (3)	0.014 (3)

### Geometric parameters (Å, °)

**Table:**

Cl1—C3	1.806 (6)	C3—C2	1.520 (8)
Cl2—C6	1.806 (6)	C3—C4	1.518 (7)
O1—C1	1.434 (8)	C3—H3	0.93 (7)
O1—C7	1.443 (7)	C4—C5	1.522 (8)
O2—C5	1.413 (7)	C4—H41	0.9700
O2—H2B	0.853 (5)	C4—H42	0.9700
O2—H2A	0.82 (2)	C5—H5	0.95 (5)
C1—C7	1.447 (9)	C6—C5	1.497 (8)
C1—H1	0.9800	C6—C7	1.497 (8)
C2—C1	1.521 (8)	C6—H6	0.88 (6)
C2—H21	0.9700	C7—H7	0.9800
C2—H22	0.9700		
			
C5—O2—H2B	124.0 (5)	C3—C4—H41	107.7
C5—O2—H2A	109 (10)	C3—C4—H42	107.7
H2B—O2—H2A	125 (10)	C5—C4—H41	107.7
C1—O1—C7	60.4 (4)	C5—C4—H42	107.7
O1—C1—C2	117.3 (6)	H42—C4—H41	107.1
O1—C1—C7	60.1 (4)	O2—C5—C4	106.1 (5)
O1—C1—H1	115.7	O2—C5—C6	112.3 (5)
C2—C1—H1	115.7	O2—C5—H5	109 (3)
C7—C1—C2	120.7 (5)	C4—C5—H5	110 (3)
C7—C1—H1	115.7	C6—C5—C4	112.9 (5)
C1—C2—H21	110.4	C6—C5—H5	107 (3)
C1—C2—H22	110.4	Cl2—C6—H6	108 (4)
C3—C2—C1	106.6 (5)	C5—C6—Cl2	111.6 (4)
C3—C2—H21	110.4	C5—C6—C7	113.6 (5)
C3—C2—H22	110.4	C5—C6—H6	116 (4)
H21—C2—H22	108.6	C7—C6—Cl2	106.3 (4)
Cl1—C3—H3	104 (4)	C7—C6—H6	101 (4)
C2—C3—Cl1	109.6 (4)	O1—C7—C1	59.5 (4)
C2—C3—H3	108 (4)	O1—C7—C6	117.4 (6)
C4—C3—Cl1	107.7 (4)	O1—C7—H7	115.4
C4—C3—C2	114.6 (5)	C1—C7—C6	122.0 (5)
C4—C3—H3	113 (4)	C1—C7—H7	115.4
C3—C4—C5	118.4 (5)	C6—C7—H7	115.4
			
C7—O1—C1—C2	−111.5 (6)	C3—C4—C5—O2	177.8 (5)
C1—O1—C7—C6	112.7 (6)	C3—C4—C5—C6	−58.7 (7)
O1—C1—C7—C6	−105.2 (7)	Cl2—C6—C5—O2	−44.3 (6)
C2—C1—C7—O1	105.9 (8)	Cl2—C6—C5—C4	−164.2 (4)
C2—C1—C7—C6	0.7 (10)	C7—C6—C5—O2	−164.5 (5)
C3—C2—C1—O1	135.8 (5)	C7—C6—C5—C4	75.6 (7)
C3—C2—C1—C7	66.0 (8)	Cl2—C6—C7—C1	168.9 (6)
Cl1—C3—C2—C1	154.6 (5)	Cl2—C6—C7—O1	99.4 (5)
C4—C3—C2—C1	−84.1 (7)	C5—C6—C7—O1	−137.5 (5)
Cl1—C3—C4—C5	−170.8 (4)	C5—C6—C7—C1	−68.0 (8)
C2—C3—C4—C5	66.8 (7)		

### Hydrogen-bond geometry (Å, °)

**Table:**

*D*—H···*A*	*D*—H	H···*A*	*D*···*A*	*D*—H···*A*
O2—H2A···O2^i^	0.83 (11)	1.96 (11)	2.746 (7)	159 (12)
O2—H2B···O2^ii^	0.85	1.84	2.692 (8)	174
C2—H21···O1^iii^	0.97	2.43	3.398 (7)	172

Symmetry codes: (i) −*x*+1, −*y*+2, −*z*; (ii) −*x*+1, *y*, −*z*+1/2; (iii) −*x*+3/2, *y*−1/2, *z*.
